# Crystal structure of nuarimol

**DOI:** 10.1107/S2056989015013493

**Published:** 2015-07-22

**Authors:** Gihaeng Kang, Jineun Kim, Hyunjin Park, Tae Ho Kim

**Affiliations:** a Department of Chemistry and Research Institute of Natural Sciences, Gyeongsang National University, Jinju 660-701, Republic of Korea

**Keywords:** crystal structure, nuarimol, pyrimidine fungicide, hydrogen bonding

## Abstract

The title compound [systematic name: (*RS*)-(2-chloro­phen­yl)(4-fluoro­phen­yl)(pyrimidin-5-yl)methanol], C_17_H_12_ClFN_2_O, is a pyrimidine fungicide. The asymmetric unit comprises two independent mol­ecules, *A* and *B*, in which the dihedral angles between the plane of the pyrimidine ring and those of the chloro­phenyl and fluoro­phenyl rings are 71.10 (6) and 70.04 (5)° in mol­ecule *A*, and 73.24 (5) and 89.30 (5)° in mol­ecule *B*. In the crystal, O—H⋯N hydrogen bonds link the components into [010] chains of alternating *A* and *B* mol­ecules. The chains are cross-linked by C—H⋯F hydrogen bonds and weak C—H⋯π and C—Cl⋯π [Cl⋯ring centroid = 3.7630 (8) Å] inter­actions, generating a three-dimensional network.

## Related literature   

For information on the fungicidal properties of the title compound, see: Demirci *et al.* (2011 ▸). For related crystal structures, see: Albinati *et al.* (1988 ▸); Caruso & Rossi (1998 ▸).
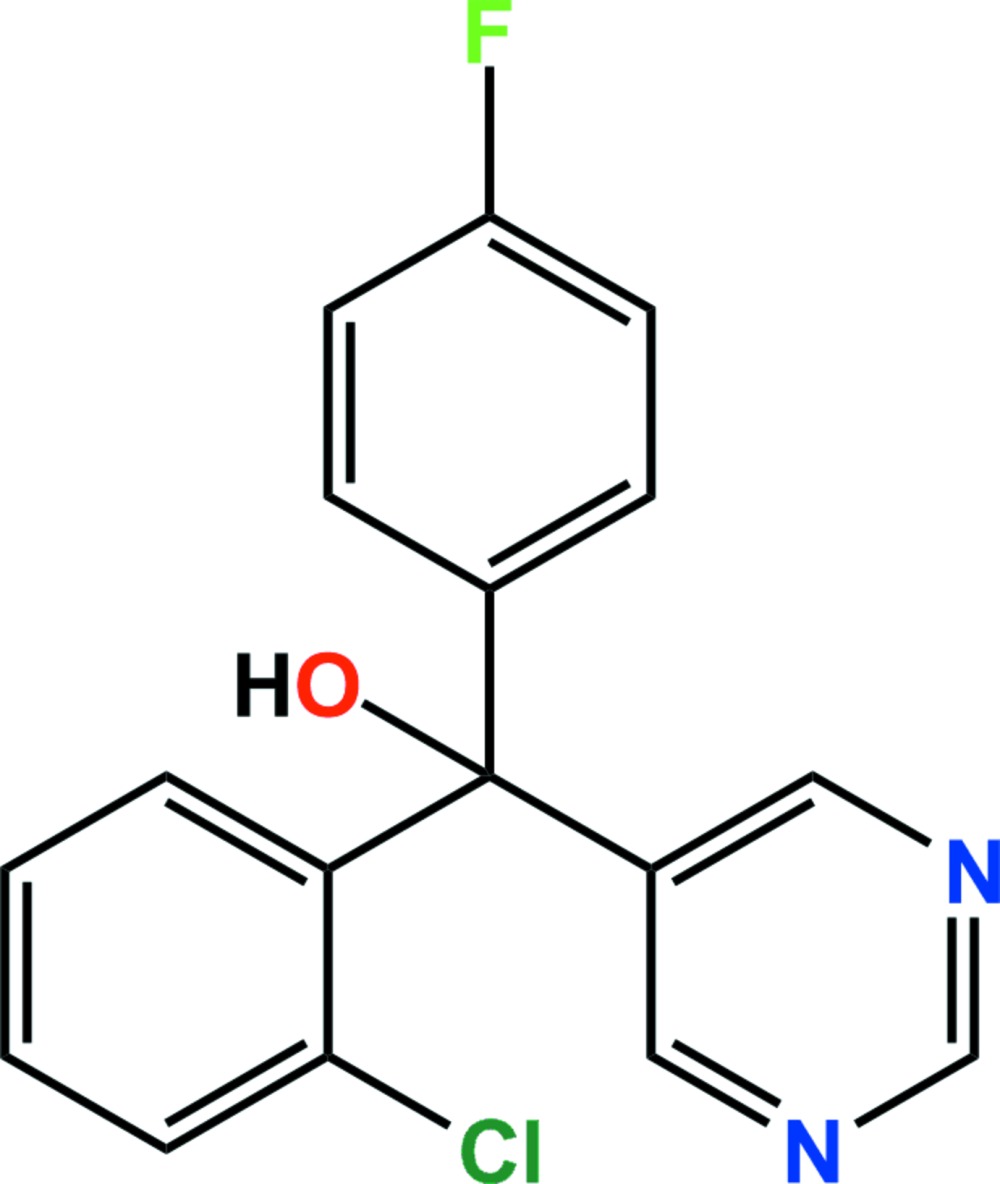


## Experimental   

### Crystal data  

C_17_H_12_ClFN_2_O
*M* *_r_* = 314.74
Monoclinic, 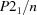

*a* = 13.5772 (5) Å
*b* = 9.3722 (4) Å
*c* = 22.8756 (10) Å
β = 99.974 (2)°
*V* = 2866.9 (2) Å^3^
*Z* = 8
Mo *K*α radiation
μ = 0.28 mm^−1^
*T* = 173 K
0.23 × 0.19 × 0.02 mm

### Data collection  

Bruker APEXII CCD diffractometer
Absorption correction: multi-scan (*SADABS*; Bruker, 2013 ▸) *T*_min_ = 0.938, *T*_max_ = 0.994
49181 measured reflections
6552 independent reflections
5243 reflections with *I* > 2σ(*I*)
*R*_int_ = 0.048

### Refinement  

*R*[*F* ^2^ > 2σ(*F* ^2^)] = 0.038
*wR*(*F* ^2^) = 0.094
*S* = 1.02
6552 reflections
399 parameters
H-atom parameters constrained
Δρ_max_ = 0.30 e Å^−3^
Δρ_min_ = −0.33 e Å^−3^

### 

Data collection: *APEX2* (Bruker, 2013 ▸); cell refinement: *SAINT* (Bruker, 2013 ▸); data reduction: *SAINT*; program(s) used to solve structure: *SHELXS97* (Sheldrick 2008 ▸); program(s) used to refine structure: *SHELXL2013* (Sheldrick, 2015 ▸); molecular graphics: *DIAMOND* (Brandenburg, 2010 ▸); software used to prepare material for publication: *SHELXTL* (Sheldrick, 2008 ▸).

## Supplementary Material

Crystal structure: contains datablock(s) global, I. DOI: 10.1107/S2056989015013493/hb7465sup1.cif

Structure factors: contains datablock(s) I. DOI: 10.1107/S2056989015013493/hb7465Isup2.hkl

Supporting information file. DOI: 10.1107/S2056989015013493/hb7465Isup3.cml

**Fig. 1.**
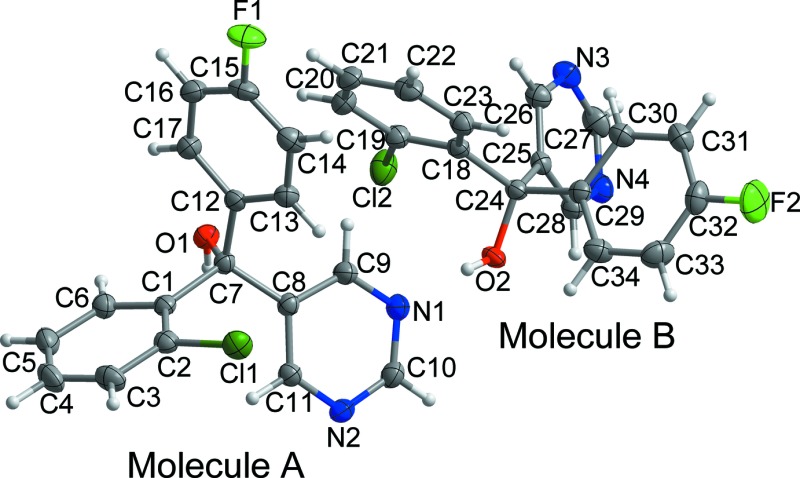
The asymmetric unit of the title compound with displacement ellipsoids drawn at the 50% probability level. H atoms are shown as small spheres of arbitrary radius.

**Fig. 2.**
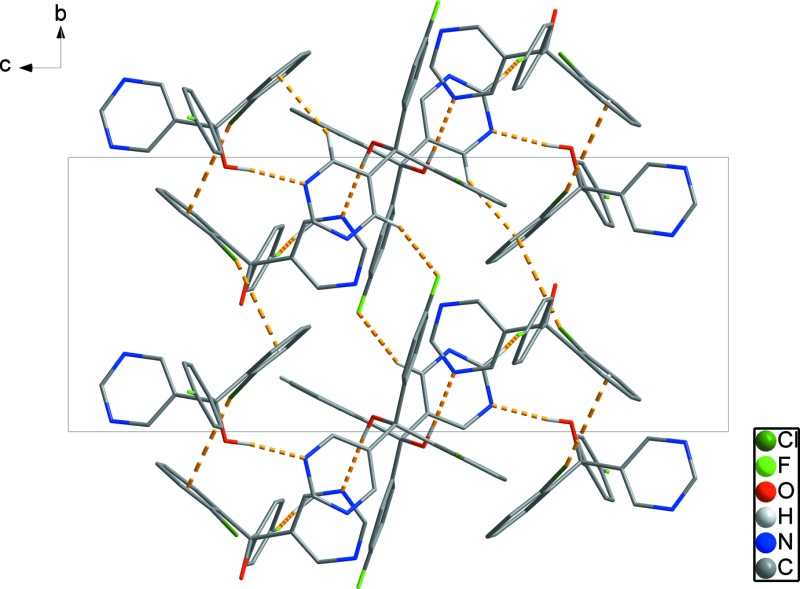
Crystal packing viewed along the a axis. The intermolecular interactions are shown as dashed lines.

. DOI: 10.1107/S2056989015013493/hb7465fig1.tif
The asymmetric unit of the title compound with displacement ellipsoids drawn at the 50% probability level. H atoms are shown as small spheres of arbitrary radius.

a . DOI: 10.1107/S2056989015013493/hb7465fig2.tif
Crystal packing viewed along the *a* axis. The inter­molecular inter­actions are shown as dashed lines.

CCDC reference: 1412613

Additional supporting information:  crystallographic information; 3D view; checkCIF report

## Figures and Tables

**Table 1:** Hydrogen-bond geometry (, ) *Cg*1 is the centroid of the C18C23 ring.

*D*H*A*	*D*H	H*A*	*D* *A*	*D*H*A*
O1H1N3^i^	0.84	2.05	2.8876(17)	176
O2H2N1	0.84	1.95	2.7807(16)	169
C10H10F2^ii^	0.95	2.33	3.0247(19)	130
C11H11F1^iii^	0.95	2.49	3.1683(18)	128
C9H9*Cg*1	0.95	2.60	3.4568(17)	149

Symmetry codes: (i) 
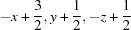
; (ii) 
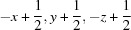
; (iii) 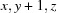.

## supplementary crystallographic information

### S1. Comment

Nuarimol, (*RS*)-2-chloro-4'-fluoro-α-(pyrimidin-5-yl)benzhydryl alcohol, is a systemic pyrimidine fungicide, which has an effect on both conidial germination and mycelial growth (Demirci *et al.*, 2011). However, until now its crystal structure has not been reported. In the title compound (Fig. 1), dihedral angles between the planes of the pyrimidine ring and the chlorophenyl and fluorophenyl ring planes are 71.10 (6), 70.04 (5) in A, 73.24 (5), and 89.30 (5)° in B, respectively. All bond lengths and bond angles are normal and comparable to those observed in similar crystal structures (Albinati *et al.*, 1988; Caruso *et al.*, 1998).

In the crystal structure (Fig. 2), O–H···N and C–H···F hydrogen bonds and weak C–H···*Cg*1 interactions are observed (Table 1). In addition, weak intermolecular C19–Cl2···*Cg*1^i^ (*Cg*1 is the centroid of the C18–C23 ring) interaction with a chlorophenyl ring is present [for symmetry code: (i), -*x* + 3/2, *y* + 1/2, -*z* + 1/2]. A three-dimensional network is formed by the hydrogen bond and these interactions.

### S2. Experimental

The title compound was purchased from the Dr Ehrenstorfer GmbH Company. Slow evaporation of a solution in CH_3_CN gave single crystals suitable for X-ray analysis in the form of colourless blocks.

### S3. Refinement

All H-atoms were positioned geometrically and refined using a riding model with d(O—H) = 0.84 Å, *U*_iso_ = 1.2*U*_eq_(C) for O—H group and d(C—H) = 0.95 Å, *U*_iso_ = 1.2*U*_eq_(C) for aromatic C—H.

### Crystal data

**Table:**

C_17_H_12_ClFN_2_O	*F*(000) = 1296
*M**_r_* = 314.74	*D*_x_ = 1.458 Mg m^−^^3^
Monoclinic, *P*2_1_/*n*	Mo *K*α radiation, λ = 0.71073 Å
*a* = 13.5772 (5) Å	Cell parameters from 9868 reflections
*b* = 9.3722 (4) Å	θ = 2.4–27.4°
*c* = 22.8756 (10) Å	µ = 0.28 mm^−^^1^
β = 99.974 (2)°	*T* = 173 K
*V* = 2866.9 (2) Å^3^	Block, colourless
*Z* = 8	0.23 × 0.19 × 0.02 mm

### Data collection

**Table:**

Bruker APEXII CCD diffractometer	5243 reflections with *I* > 2σ(*I*)
φ and ω scans	*R*_int_ = 0.048
Absorption correction: multi-scan (*SADABS*; Bruker, 2013)	θ_max_ = 27.5°, θ_min_ = 1.6°
*T*_min_ = 0.938, *T*_max_ = 0.994	*h* = −17→15
49181 measured reflections	*k* = −12→12
6552 independent reflections	*l* = −26→29

### Refinement

**Table:**

Refinement on *F*^2^	0 restraints
Least-squares matrix: full	Hydrogen site location: inferred from neighbouring sites
*R*[*F*^2^ > 2σ(*F*^2^)] = 0.038	H-atom parameters constrained
*wR*(*F*^2^) = 0.094	*w* = 1/[σ^2^(*F*_o_^2^) + (0.0373*P*)^2^ + 1.2482*P*] where *P* = (*F*_o_^2^ + 2*F*_c_^2^)/3
*S* = 1.02	(Δ/σ)_max_ = 0.001
6552 reflections	Δρ_max_ = 0.30 e Å^−^^3^
399 parameters	Δρ_min_ = −0.33 e Å^−^^3^

### Special details

**Table:**

Geometry. All e.s.d.'s (except the e.s.d. in the dihedral angle between two l.s. planes) are estimated using the full covariance matrix. The cell e.s.d.'s are taken into account individually in the estimation of e.s.d.'s in distances, angles and torsion angles; correlations between e.s.d.'s in cell parameters are only used when they are defined by crystal symmetry. An approximate (isotropic) treatment of cell e.s.d.'s is used for estimating e.s.d.'s involving l.s. planes.

### Fractional atomic coordinates and isotropic or equivalent isotropic displacement parameters (Å^2^)

**Table:**

	*x*	*y*	*z*	*U*_iso_*/*U*_eq_	
Cl1	0.63147 (3)	1.08339 (5)	0.58828 (2)	0.03329 (11)	
Cl2	0.76439 (3)	0.87663 (5)	0.24544 (2)	0.04037 (12)	
F1	0.63952 (9)	0.43385 (10)	0.56060 (5)	0.0435 (3)	
F2	0.10747 (8)	0.85446 (14)	0.18266 (6)	0.0578 (3)	
O1	0.84803 (8)	0.94613 (11)	0.46184 (5)	0.0252 (2)	
H1	0.8638	1.0227	0.4467	0.038*	
O2	0.55752 (8)	1.03372 (10)	0.23794 (4)	0.0252 (2)	
H2	0.5641	1.0449	0.2748	0.038*	
N1	0.59896 (11)	1.09694 (14)	0.35847 (6)	0.0306 (3)	
N2	0.63234 (10)	1.29702 (14)	0.42266 (6)	0.0290 (3)	
N3	0.59877 (10)	0.71612 (15)	0.08495 (6)	0.0307 (3)	
N4	0.56029 (11)	0.96125 (15)	0.06519 (6)	0.0328 (3)	
C1	0.81585 (11)	1.04686 (15)	0.55498 (6)	0.0215 (3)	
C2	0.76148 (12)	1.09722 (16)	0.59716 (7)	0.0249 (3)	
C3	0.80810 (14)	1.16167 (18)	0.64950 (7)	0.0347 (4)	
H3	0.7691	1.1970	0.6770	0.042*	
C4	0.91094 (15)	1.17431 (19)	0.66147 (8)	0.0396 (4)	
H4	0.9429	1.2177	0.6973	0.048*	
C5	0.96701 (14)	1.12369 (18)	0.62112 (8)	0.0346 (4)	
H5	1.0378	1.1312	0.6292	0.041*	
C6	0.91950 (12)	1.06180 (16)	0.56867 (7)	0.0273 (3)	
H6	0.9590	1.0284	0.5411	0.033*	
C7	0.77056 (11)	0.97330 (15)	0.49555 (6)	0.0214 (3)	
C8	0.69528 (11)	1.06668 (15)	0.45545 (6)	0.0212 (3)	
C9	0.64915 (12)	1.01376 (17)	0.40093 (7)	0.0278 (3)	
H9	0.6534	0.9144	0.3935	0.033*	
C10	0.59480 (12)	1.23530 (17)	0.37161 (7)	0.0299 (4)	
H10	0.5614	1.2958	0.3412	0.036*	
C11	0.68292 (12)	1.21098 (16)	0.46406 (7)	0.0249 (3)	
H11	0.7117	1.2512	0.5012	0.030*	
C12	0.73077 (11)	0.82590 (15)	0.50925 (6)	0.0218 (3)	
C13	0.62998 (12)	0.79740 (16)	0.50815 (7)	0.0263 (3)	
H13	0.5819	0.8698	0.4959	0.032*	
C14	0.59850 (13)	0.66483 (17)	0.52473 (7)	0.0310 (4)	
H14	0.5296	0.6455	0.5241	0.037*	
C15	0.66972 (14)	0.56261 (16)	0.54209 (7)	0.0303 (4)	
C16	0.76940 (13)	0.58404 (17)	0.54206 (7)	0.0311 (4)	
H16	0.8165	0.5096	0.5531	0.037*	
C17	0.80007 (12)	0.71699 (16)	0.52556 (7)	0.0274 (3)	
H17	0.8690	0.7341	0.5254	0.033*	
C18	0.58321 (11)	0.78920 (15)	0.27131 (6)	0.0223 (3)	
C19	0.68755 (12)	0.77899 (16)	0.28450 (7)	0.0265 (3)	
C20	0.73552 (13)	0.69367 (17)	0.33029 (7)	0.0322 (4)	
H20	0.8064	0.6877	0.3378	0.039*	
C21	0.68046 (14)	0.61731 (17)	0.36501 (7)	0.0325 (4)	
H21	0.7133	0.5594	0.3965	0.039*	
C22	0.57749 (14)	0.62565 (17)	0.35365 (7)	0.0314 (4)	
H22	0.5391	0.5736	0.3774	0.038*	
C23	0.52998 (12)	0.71057 (16)	0.30732 (7)	0.0265 (3)	
H23	0.4590	0.7152	0.3000	0.032*	
C24	0.52929 (11)	0.89043 (15)	0.22322 (6)	0.0212 (3)	
C25	0.55638 (11)	0.86464 (15)	0.16185 (6)	0.0216 (3)	
C26	0.58502 (12)	0.73512 (17)	0.14135 (7)	0.0272 (3)	
H26	0.5954	0.6566	0.1680	0.033*	
C27	0.58488 (13)	0.83070 (19)	0.05016 (7)	0.0331 (4)	
H27	0.5935	0.8178	0.0102	0.040*	
C28	0.54549 (12)	0.97556 (17)	0.12101 (7)	0.0262 (3)	
H28	0.5265	1.0666	0.1335	0.031*	
C29	0.41437 (11)	0.87642 (15)	0.21441 (6)	0.0213 (3)	
C30	0.36495 (12)	0.75843 (17)	0.18589 (7)	0.0282 (3)	
H30	0.4030	0.6824	0.1736	0.034*	
C31	0.26178 (13)	0.75007 (18)	0.17516 (7)	0.0318 (4)	
H31	0.2286	0.6692	0.1559	0.038*	
C32	0.20876 (12)	0.86111 (19)	0.19289 (7)	0.0337 (4)	
C33	0.25363 (13)	0.97840 (19)	0.22114 (8)	0.0384 (4)	
H33	0.2147	1.0538	0.2331	0.046*	
C34	0.35716 (13)	0.98521 (18)	0.23203 (7)	0.0312 (4)	
H34	0.3894	1.0660	0.2519	0.037*	

### Atomic displacement parameters (Å^2^)

**Table:**

	*U*^11^	*U*^22^	*U*^33^	*U*^12^	*U*^13^	*U*^23^
Cl1	0.0317 (2)	0.0379 (2)	0.0325 (2)	0.00085 (17)	0.01153 (17)	−0.00473 (17)
Cl2	0.0281 (2)	0.0486 (3)	0.0437 (3)	−0.00338 (19)	0.00418 (18)	0.0138 (2)
F1	0.0620 (7)	0.0226 (5)	0.0485 (6)	−0.0083 (5)	0.0167 (5)	0.0002 (4)
F2	0.0242 (6)	0.0786 (9)	0.0697 (8)	0.0000 (6)	0.0055 (5)	−0.0044 (7)
O1	0.0256 (6)	0.0270 (6)	0.0240 (6)	0.0028 (5)	0.0070 (4)	0.0001 (4)
O2	0.0362 (6)	0.0189 (5)	0.0183 (5)	−0.0040 (4)	−0.0011 (5)	−0.0018 (4)
N1	0.0362 (8)	0.0295 (7)	0.0226 (7)	0.0029 (6)	−0.0042 (6)	−0.0005 (6)
N2	0.0334 (8)	0.0243 (7)	0.0278 (7)	0.0059 (6)	0.0010 (6)	0.0006 (6)
N3	0.0335 (8)	0.0334 (8)	0.0267 (7)	−0.0027 (6)	0.0089 (6)	−0.0052 (6)
N4	0.0407 (8)	0.0354 (8)	0.0210 (7)	−0.0037 (6)	0.0019 (6)	0.0014 (6)
C1	0.0261 (8)	0.0173 (7)	0.0203 (7)	0.0007 (6)	0.0013 (6)	0.0026 (6)
C2	0.0300 (8)	0.0215 (7)	0.0230 (8)	−0.0008 (6)	0.0037 (6)	0.0014 (6)
C3	0.0491 (11)	0.0319 (9)	0.0230 (8)	−0.0022 (8)	0.0064 (8)	−0.0061 (7)
C4	0.0514 (12)	0.0354 (10)	0.0270 (9)	−0.0085 (8)	−0.0072 (8)	−0.0073 (7)
C5	0.0325 (9)	0.0314 (9)	0.0353 (9)	−0.0058 (7)	−0.0067 (7)	−0.0004 (7)
C6	0.0281 (8)	0.0265 (8)	0.0257 (8)	−0.0007 (6)	0.0004 (6)	0.0010 (6)
C7	0.0216 (7)	0.0227 (7)	0.0192 (7)	0.0027 (6)	0.0021 (6)	−0.0012 (6)
C8	0.0223 (7)	0.0227 (7)	0.0184 (7)	0.0016 (6)	0.0032 (6)	0.0005 (6)
C9	0.0341 (9)	0.0221 (8)	0.0251 (8)	0.0023 (7)	−0.0005 (7)	−0.0013 (6)
C10	0.0308 (9)	0.0303 (9)	0.0266 (8)	0.0064 (7)	−0.0012 (7)	0.0030 (7)
C11	0.0283 (8)	0.0240 (8)	0.0216 (7)	0.0017 (6)	0.0025 (6)	−0.0018 (6)
C12	0.0262 (8)	0.0203 (7)	0.0177 (7)	0.0010 (6)	0.0008 (6)	−0.0032 (6)
C13	0.0267 (8)	0.0245 (8)	0.0272 (8)	0.0019 (6)	0.0030 (6)	−0.0026 (6)
C14	0.0310 (9)	0.0292 (9)	0.0341 (9)	−0.0055 (7)	0.0090 (7)	−0.0070 (7)
C15	0.0459 (10)	0.0188 (8)	0.0269 (8)	−0.0057 (7)	0.0087 (7)	−0.0043 (6)
C16	0.0380 (10)	0.0221 (8)	0.0316 (9)	0.0052 (7)	0.0013 (7)	−0.0005 (7)
C17	0.0273 (8)	0.0248 (8)	0.0287 (8)	0.0025 (6)	0.0005 (7)	−0.0017 (6)
C18	0.0288 (8)	0.0189 (7)	0.0184 (7)	0.0000 (6)	0.0013 (6)	−0.0017 (6)
C19	0.0300 (8)	0.0230 (8)	0.0259 (8)	−0.0008 (6)	0.0031 (6)	0.0015 (6)
C20	0.0322 (9)	0.0265 (8)	0.0347 (9)	0.0057 (7)	−0.0035 (7)	0.0001 (7)
C21	0.0441 (10)	0.0227 (8)	0.0273 (8)	0.0053 (7)	−0.0033 (7)	0.0036 (6)
C22	0.0458 (10)	0.0239 (8)	0.0239 (8)	−0.0028 (7)	0.0045 (7)	0.0048 (6)
C23	0.0303 (8)	0.0253 (8)	0.0231 (8)	−0.0022 (7)	0.0023 (6)	0.0000 (6)
C24	0.0269 (8)	0.0176 (7)	0.0183 (7)	−0.0012 (6)	0.0015 (6)	−0.0009 (5)
C25	0.0207 (7)	0.0235 (7)	0.0194 (7)	−0.0028 (6)	0.0006 (6)	−0.0014 (6)
C26	0.0317 (9)	0.0261 (8)	0.0242 (8)	−0.0015 (7)	0.0060 (7)	−0.0005 (6)
C27	0.0374 (10)	0.0417 (10)	0.0210 (8)	−0.0070 (8)	0.0076 (7)	−0.0043 (7)
C28	0.0298 (8)	0.0253 (8)	0.0217 (8)	−0.0010 (6)	−0.0003 (6)	0.0005 (6)
C29	0.0267 (8)	0.0213 (7)	0.0154 (7)	0.0012 (6)	0.0023 (6)	0.0027 (6)
C30	0.0304 (9)	0.0261 (8)	0.0275 (8)	−0.0005 (7)	0.0037 (7)	−0.0046 (6)
C31	0.0309 (9)	0.0358 (9)	0.0272 (8)	−0.0071 (7)	0.0010 (7)	−0.0041 (7)
C32	0.0236 (8)	0.0467 (10)	0.0305 (9)	0.0011 (7)	0.0040 (7)	0.0064 (8)
C33	0.0355 (10)	0.0344 (10)	0.0483 (11)	0.0073 (8)	0.0155 (8)	−0.0017 (8)
C34	0.0346 (9)	0.0267 (8)	0.0337 (9)	−0.0008 (7)	0.0096 (7)	−0.0057 (7)

### Geometric parameters (Å, º)

**Table:**

Cl1—C2	1.7464 (16)	C13—C14	1.388 (2)
Cl2—C19	1.7456 (16)	C13—H13	0.9500
F1—C15	1.3653 (18)	C14—C15	1.370 (2)
F2—C32	1.3560 (19)	C14—H14	0.9500
O1—C7	1.4307 (17)	C15—C16	1.368 (2)
O1—H1	0.8400	C16—C17	1.387 (2)
O2—C24	1.4209 (17)	C16—H16	0.9500
O2—H2	0.8400	C17—H17	0.9500
N1—C10	1.335 (2)	C18—C23	1.397 (2)
N1—C9	1.337 (2)	C18—C19	1.400 (2)
N2—C10	1.324 (2)	C18—C24	1.538 (2)
N2—C11	1.3392 (19)	C19—C20	1.387 (2)
N3—C27	1.331 (2)	C20—C21	1.381 (2)
N3—C26	1.347 (2)	C20—H20	0.9500
N4—C27	1.329 (2)	C21—C22	1.379 (3)
N4—C28	1.333 (2)	C21—H21	0.9500
C1—C6	1.395 (2)	C22—C23	1.391 (2)
C1—C2	1.395 (2)	C22—H22	0.9500
C1—C7	1.553 (2)	C23—H23	0.9500
C2—C3	1.392 (2)	C24—C25	1.531 (2)
C3—C4	1.381 (3)	C24—C29	1.544 (2)
C3—H3	0.9500	C25—C26	1.381 (2)
C4—C5	1.378 (3)	C25—C28	1.388 (2)
C4—H4	0.9500	C26—H26	0.9500
C5—C6	1.387 (2)	C27—H27	0.9500
C5—H5	0.9500	C28—H28	0.9500
C6—H6	0.9500	C29—C34	1.383 (2)
C7—C8	1.526 (2)	C29—C30	1.395 (2)
C7—C12	1.535 (2)	C30—C31	1.382 (2)
C8—C11	1.381 (2)	C30—H30	0.9500
C8—C9	1.387 (2)	C31—C32	1.366 (2)
C9—H9	0.9500	C31—H31	0.9500
C10—H10	0.9500	C32—C33	1.364 (3)
C11—H11	0.9500	C33—C34	1.386 (2)
C12—C13	1.390 (2)	C33—H33	0.9500
C12—C17	1.395 (2)	C34—H34	0.9500
			
C7—O1—H1	109.5	C16—C17—C12	120.66 (15)
C24—O2—H2	109.5	C16—C17—H17	119.7
C10—N1—C9	116.05 (13)	C12—C17—H17	119.7
C10—N2—C11	115.64 (13)	C23—C18—C19	116.40 (14)
C27—N3—C26	115.93 (14)	C23—C18—C24	121.09 (14)
C27—N4—C28	115.25 (14)	C19—C18—C24	122.33 (13)
C6—C1—C2	116.42 (14)	C20—C19—C18	121.84 (15)
C6—C1—C7	118.18 (13)	C20—C19—Cl2	116.40 (13)
C2—C1—C7	125.38 (13)	C18—C19—Cl2	121.75 (12)
C3—C2—C1	121.72 (15)	C21—C20—C19	120.19 (16)
C3—C2—Cl1	115.60 (13)	C21—C20—H20	119.9
C1—C2—Cl1	122.68 (12)	C19—C20—H20	119.9
C4—C3—C2	120.09 (16)	C22—C21—C20	119.62 (15)
C4—C3—H3	120.0	C22—C21—H21	120.2
C2—C3—H3	120.0	C20—C21—H21	120.2
C5—C4—C3	119.64 (15)	C21—C22—C23	119.81 (16)
C5—C4—H4	120.2	C21—C22—H22	120.1
C3—C4—H4	120.2	C23—C22—H22	120.1
C4—C5—C6	119.68 (17)	C22—C23—C18	122.15 (15)
C4—C5—H5	120.2	C22—C23—H23	118.9
C6—C5—H5	120.2	C18—C23—H23	118.9
C5—C6—C1	122.42 (16)	O2—C24—C25	105.59 (11)
C5—C6—H6	118.8	O2—C24—C18	109.76 (11)
C1—C6—H6	118.8	C25—C24—C18	113.46 (12)
O1—C7—C8	104.86 (11)	O2—C24—C29	109.65 (12)
O1—C7—C12	105.51 (11)	C25—C24—C29	105.35 (11)
C8—C7—C12	114.59 (12)	C18—C24—C29	112.69 (12)
O1—C7—C1	109.53 (12)	C26—C25—C28	115.66 (14)
C8—C7—C1	113.22 (12)	C26—C25—C24	125.21 (13)
C12—C7—C1	108.71 (11)	C28—C25—C24	118.97 (13)
C11—C8—C9	115.56 (14)	N3—C26—C25	122.36 (14)
C11—C8—C7	124.14 (13)	N3—C26—H26	118.8
C9—C8—C7	119.13 (13)	C25—C26—H26	118.8
N1—C9—C8	122.61 (14)	N4—C27—N3	127.17 (15)
N1—C9—H9	118.7	N4—C27—H27	116.4
C8—C9—H9	118.7	N3—C27—H27	116.4
N2—C10—N1	126.77 (14)	N4—C28—C25	123.58 (15)
N2—C10—H10	116.6	N4—C28—H28	118.2
N1—C10—H10	116.6	C25—C28—H28	118.2
N2—C11—C8	123.29 (14)	C34—C29—C30	118.14 (15)
N2—C11—H11	118.4	C34—C29—C24	120.33 (13)
C8—C11—H11	118.4	C30—C29—C24	121.44 (13)
C13—C12—C17	118.72 (14)	C31—C30—C29	121.23 (15)
C13—C12—C7	123.32 (13)	C31—C30—H30	119.4
C17—C12—C7	117.92 (14)	C29—C30—H30	119.4
C14—C13—C12	121.05 (15)	C32—C31—C30	118.30 (15)
C14—C13—H13	119.5	C32—C31—H31	120.9
C12—C13—H13	119.5	C30—C31—H31	120.9
C15—C14—C13	118.02 (16)	F2—C32—C33	118.37 (16)
C15—C14—H14	121.0	F2—C32—C31	118.98 (16)
C13—C14—H14	121.0	C33—C32—C31	122.65 (16)
F1—C15—C16	118.70 (15)	C32—C33—C34	118.56 (16)
F1—C15—C14	118.23 (16)	C32—C33—H33	120.7
C16—C15—C14	123.07 (15)	C34—C33—H33	120.7
C15—C16—C17	118.41 (15)	C29—C34—C33	121.12 (15)
C15—C16—H16	120.8	C29—C34—H34	119.4
C17—C16—H16	120.8	C33—C34—H34	119.4
			
C6—C1—C2—C3	1.3 (2)	C23—C18—C19—C20	1.0 (2)
C7—C1—C2—C3	179.80 (14)	C24—C18—C19—C20	176.13 (14)
C6—C1—C2—Cl1	−178.40 (11)	C23—C18—C19—Cl2	−177.32 (11)
C7—C1—C2—Cl1	0.1 (2)	C24—C18—C19—Cl2	−2.2 (2)
C1—C2—C3—C4	−1.5 (2)	C18—C19—C20—C21	−1.0 (2)
Cl1—C2—C3—C4	178.29 (13)	Cl2—C19—C20—C21	177.38 (13)
C2—C3—C4—C5	0.4 (3)	C19—C20—C21—C22	0.4 (2)
C3—C4—C5—C6	0.6 (3)	C20—C21—C22—C23	0.1 (2)
C4—C5—C6—C1	−0.7 (3)	C21—C22—C23—C18	−0.1 (2)
C2—C1—C6—C5	−0.2 (2)	C19—C18—C23—C22	−0.4 (2)
C7—C1—C6—C5	−178.83 (14)	C24—C18—C23—C22	−175.65 (14)
C6—C1—C7—O1	−5.94 (18)	C23—C18—C24—O2	114.19 (15)
C2—C1—C7—O1	175.61 (13)	C19—C18—C24—O2	−60.75 (18)
C6—C1—C7—C8	−122.57 (14)	C23—C18—C24—C25	−127.96 (15)
C2—C1—C7—C8	58.98 (19)	C19—C18—C24—C25	57.10 (18)
C6—C1—C7—C12	108.87 (15)	C23—C18—C24—C29	−8.33 (19)
C2—C1—C7—C12	−69.58 (17)	C19—C18—C24—C29	176.73 (13)
O1—C7—C8—C11	−106.42 (16)	O2—C24—C25—C26	149.18 (14)
C12—C7—C8—C11	138.39 (15)	C18—C24—C25—C26	28.9 (2)
C1—C7—C8—C11	12.9 (2)	C29—C24—C25—C26	−94.79 (17)
O1—C7—C8—C9	60.67 (17)	O2—C24—C25—C28	−35.57 (18)
C12—C7—C8—C9	−54.52 (19)	C18—C24—C25—C28	−155.81 (13)
C1—C7—C8—C9	−179.99 (13)	C29—C24—C25—C28	80.46 (16)
C10—N1—C9—C8	−0.5 (2)	C27—N3—C26—C25	1.2 (2)
C11—C8—C9—N1	2.3 (2)	C28—C25—C26—N3	−1.8 (2)
C7—C8—C9—N1	−165.84 (15)	C24—C25—C26—N3	173.55 (14)
C11—N2—C10—N1	2.9 (3)	C28—N4—C27—N3	−2.1 (3)
C9—N1—C10—N2	−2.3 (3)	C26—N3—C27—N4	0.9 (3)
C10—N2—C11—C8	−0.7 (2)	C27—N4—C28—C25	1.2 (2)
C9—C8—C11—N2	−1.7 (2)	C26—C25—C28—N4	0.6 (2)
C7—C8—C11—N2	165.81 (15)	C24—C25—C28—N4	−175.13 (14)
O1—C7—C12—C13	−140.63 (14)	O2—C24—C29—C34	−12.53 (18)
C8—C7—C12—C13	−25.8 (2)	C25—C24—C29—C34	−125.73 (14)
C1—C7—C12—C13	101.97 (16)	C18—C24—C29—C34	110.05 (15)
O1—C7—C12—C17	41.66 (17)	O2—C24—C29—C30	164.03 (13)
C8—C7—C12—C17	156.47 (13)	C25—C24—C29—C30	50.83 (17)
C1—C7—C12—C17	−75.74 (16)	C18—C24—C29—C30	−73.39 (17)
C17—C12—C13—C14	2.0 (2)	C34—C29—C30—C31	0.3 (2)
C7—C12—C13—C14	−175.65 (14)	C24—C29—C30—C31	−176.34 (14)
C12—C13—C14—C15	−0.2 (2)	C29—C30—C31—C32	0.4 (2)
C13—C14—C15—F1	177.54 (14)	C30—C31—C32—F2	180.00 (15)
C13—C14—C15—C16	−2.0 (2)	C30—C31—C32—C33	−0.7 (3)
F1—C15—C16—C17	−177.36 (14)	F2—C32—C33—C34	179.58 (16)
C14—C15—C16—C17	2.2 (2)	C31—C32—C33—C34	0.3 (3)
C15—C16—C17—C12	−0.2 (2)	C30—C29—C34—C33	−0.7 (2)
C13—C12—C17—C16	−1.9 (2)	C24—C29—C34—C33	175.94 (15)
C7—C12—C17—C16	175.96 (14)	C32—C33—C34—C29	0.5 (3)

### Hydrogen-bond geometry (Å, º)

Cg1 is the centroid of the C18–C23 ring.

**Table:**

*D*—H···*A*	*D*—H	H···*A*	*D*···*A*	*D*—H···*A*
O1—H1···N3^i^	0.84	2.05	2.8876 (17)	176
O2—H2···N1	0.84	1.95	2.7807 (16)	169
C10—H10···F2^ii^	0.95	2.33	3.0247 (19)	130
C11—H11···F1^iii^	0.95	2.49	3.1683 (18)	128
C9—H9···*Cg*1	0.95	2.60	3.4568 (17)	149

Symmetry codes: (i) −*x*+3/2, *y*+1/2, −*z*+1/2; (ii) −*x*+1/2, *y*+1/2, −*z*+1/2; (iii) *x*, *y*+1, *z*.
