# Supplementary figures and images for: Crystal structure of nuarimol
Source: Acta Crystallogr E Crystallogr Commun. 2015 Jul 22;71(Pt 8):o586–7. doi: 10.1107/S2056989015013493 (PMC4571410; doi:10.1107/S2056989015013493)

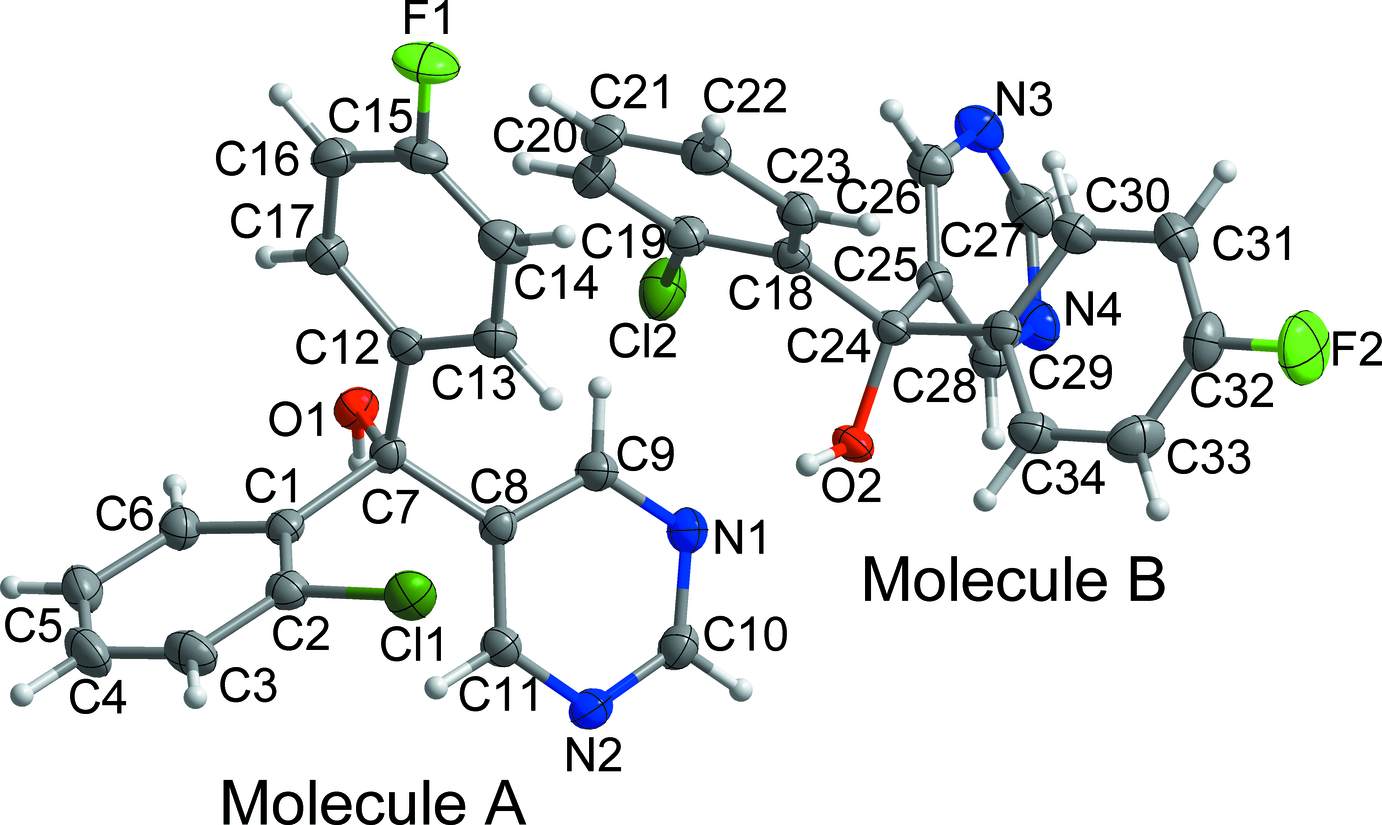

Supplement: Supplementary file 4 [file e-71-0o586-fig1.tif]

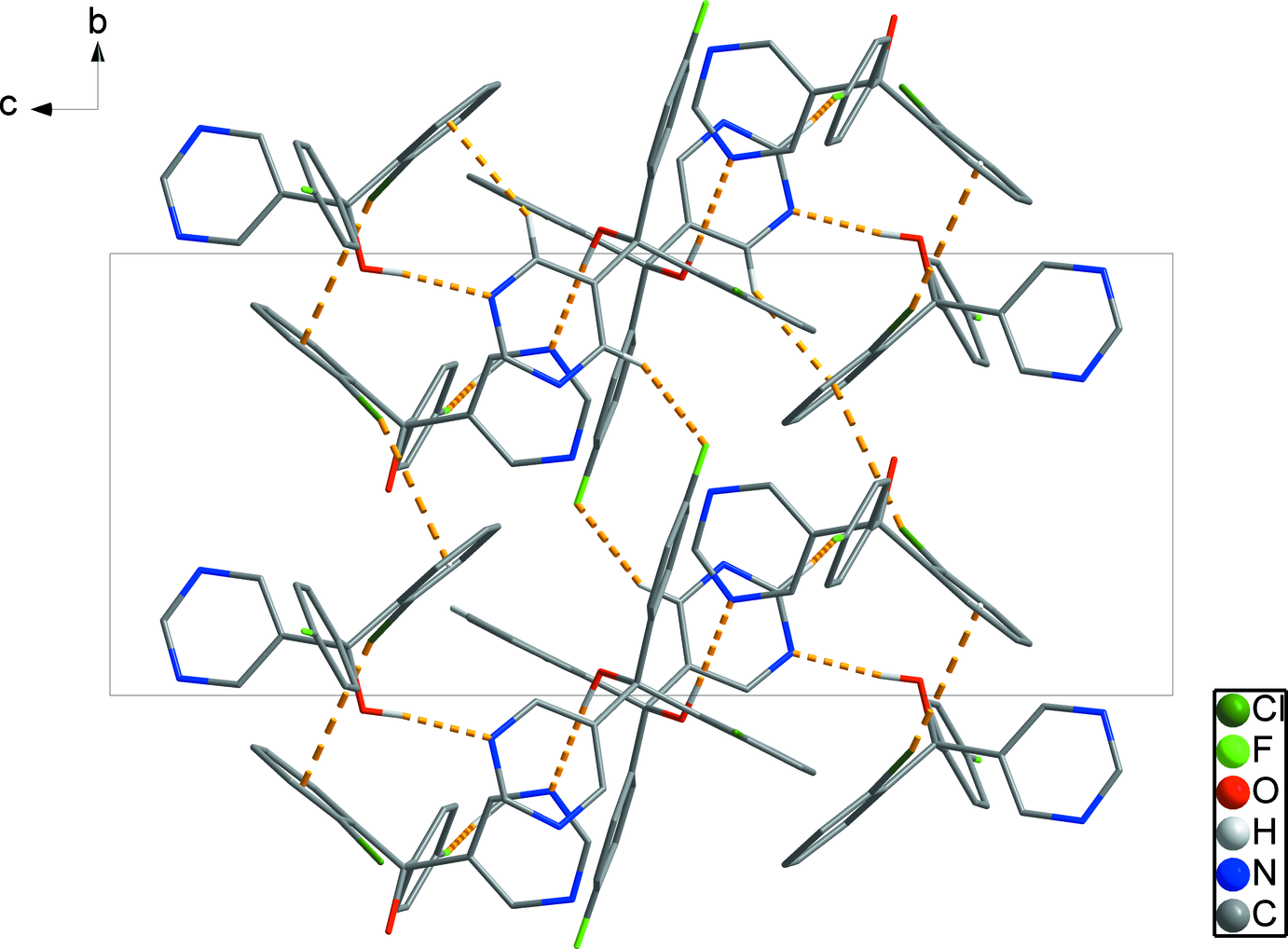

Supplement: Supplementary file 5 [file e-71-0o586-fig2.tif]
